# Beyond a waste product: lactate as a master metabolite dictating anti-tumor T-cell fate

**DOI:** 10.1038/s41419-026-08976-8

**Published:** 2026-06-23

**Authors:** Xiangyu Dai, Jingjia Chang, Siyue Wang, Lutterodt Bentum-Ennin, Jian Zhou, Xiaomin Zuo, Wanglai Hu

**Affiliations:** 1 State Key Laboratory of Metabolic Dysregulation & Prevention and Treatment of Esophageal Cancer, Tianjian Laboratory of Advanced Biomedical Sciences, School of Convergence Medicine, Henan International Joint Laboratory of Non-coding RNA and Metabolism in Cancer, People’s Hospital of Zhengzhou University, Zhengzhou University, Zhengzhou, 450003 China; 2 Department of Immunology, School of Basic Medical Sciences, Anhui Medical University, Hefei, 230027 China; 3 Department of Orthopedics, The First Affiliated Hospital of Anhui Medical University, Hefei, 230022 China; 4 Department of General Surgery, The First Affiliated Hospital of Anhui Medical University, Hefei, 230022 China

**Keywords:** Cancer metabolism, Cell biology

## Abstract

Lactate, a key byproduct of tumor metabolic reprogramming, accumulates in the tumor microenvironment (TME) and profoundly shapes T cell-mediated anti-tumor immunity. As research into TME metabolism advances, lactate has emerged as a critical regulator with broad effects on immune function. In many cancers—including gastric cancer, hepatocellular carcinoma, lung cancer, melanoma, and pancreatic cancer—lactate suppresses or remodels anti-tumor immunity by acting on CD8⁺ T cells, regulatory T cells (Tregs), dendritic cells (DCs), and immune checkpoint molecules. The underlying mechanisms are becoming increasingly well-defined. However, major knowledge gaps remain, especially regarding how lactate-associated enzymes (e.g., LDHA), lactate transporters (e.g., MCT4), and signaling pathways impact T cell function. This review summarizes how lactate regulates anti-tumor immune responses and explores emerging immunotherapies targeting lactate metabolism, with a focus on metabolic enzymes and transporters. We cover preclinical and clinical progress on LDHA inhibitors and lactate transporter inhibitors. By comprehensively analyzing lactate’s function in the TME, we aim to build a theoretical framework for precision tumor immunotherapy and propose future directions centered on modulating the immune microenvironment through lactate-targeted strategies.

## FACTS

- Lactate acts as a bioactive signaling molecule and metabolic intermediate in the TME, rather than just a glycolytic byproduct.
- High lactate levels impair CD8⁺ T cell effector function and boost the metabolic fitness and immunosuppressive activity of regulatory immune cells.
- Lactate rewires anti-tumor immunity through metabolic, transcriptional, and epigenetic mechanisms—most notably protein and histone lactylation.
- Enzymes and transporters in lactate metabolism (e.g., LDHA, MCTs) are promising targets for enhancing tumor immunotherapy.

## Introduction

Lactate is now widely recognized as a key player in the tumor microenvironment (TME). As a major byproduct of tumor metabolic reprogramming, lactate accumulation is closely linked to tumor progression and immune evasion. Recent studies have revealed that lactate is far more than a metabolic waste product. It also acts as a critical signaling molecule that regulates diverse cellular activities in the TME. Importantly, lactate plays a central role in helping tumors escape immune attack [1, 2].

In the TME, lactate mainly comes from elevated aerobic glycolysis—the well-known “Warburg effect” of cancer cells [3, 4]. Excess lactate acidifies the local environment and severely impairs immune cell function, especially that of CD8⁺ effector T cells (CD8⁺-T cells) [1, 5]. Studies have shown that lactate dampens anti-tumor immune responses by reducing immune cell activity and blocking their proliferation, which in turn promotes tumor growth and metastasis [4].

Lactate also strengthens the stability and immunosuppressive function of regulatory T cells (Tregs), amplifying the TME’s immunosuppressive characteristics [4, 6]. Specifically, lactate upregulates genes associated with immune evasion, promoting Treg accumulation in the TME, and inhibits effector T cell activity [2]. Understanding this dual role of lactate is essential for unraveling how tumors evade immune surveillance.

Therapeutic strategies targeting lactate metabolism are emerging as promising ways to boost immunotherapy efficacy. Lactate dehydrogenase (LDH) inhibitors, which reduce lactate production, show strong potential to reprogram the immunosuppressive TME and enhance anti-tumor immune responses [4, 7]. Additionally, combining lactate modulation with immune checkpoint blockade has yielded encouraging preclinical results, pointing to a new direction for cancer treatment [8].

In summary, lactate accumulation in the TME serves multiple functions. It supports tumor growth and metastasis, and it helps tumors evade immunity by impairing immune cell function. This review systematically examines how lactate shapes T cell-mediated anti-tumor immunity and highlights recent advances in therapies targeting lactate metabolism, laying a foundation for future research and clinical applications.

## Lactate production and its impact on T cell function in the TME

Under normal physiological conditions, pyruvate produced by glycolysis is fully oxidized in mitochondria through the tricarboxylic acid (TCA) cycle with ATP is generated via oxidative phosphorylation (OXPHOS) and CO₂ is produced as a byproduct [9]. In normal cells, lactate is mainly produced under hypoxia through anaerobic glycolysis. By contrast, tumor cells continuously produce large amounts of lactate even in the presence of oxygen, a hallmark called aerobic glycolysis, or the Warburg effect [10]. Lactate can activate HIF-1 in endothelial cells under normoxia, promoting the expression of bFGF and VEGFR2. This enhances VEGF signaling triggered by lactate-induced VEGF expression in tumor and stromal cells, forming a positive feedback loop [11]. Studies in mouse xenograft models of human colorectal and breast cancer have shown that lactate released by tumor cells via MCT4 (rather than MCT1) is sufficient to stimulate IL-8-dependent angiogenesis and tumor growth [12]. In addition, accumulating evidence indicates that lactate regulates the degradation of collagen IV, collagen VII, and glycoproteins, as well as the synthesis of collagen I. It affects multiple signaling pathways, including TGF-β/Smad, Wnt/β-catenin, IL-6/STAT3, and HGF/MET. These pathways contribute to basement membrane (BM) remodeling and epithelial-mesenchymal transition (EMT), ultimately driving tumor invasion [13, 14].

Although lactate accumulation gives tumor cells strong growth and survival advantages, its most profound effect in the TME is reshaping immune cell metabolism and function. Tumor cells export excess lactate into the extracellular space through monocarboxylate transporters (MCTs), especially MCT1 and MCT4, to avoid intracellular buildup. This process raises lactate levels throughout the TME [8, 15]. Notably, this lactate-driven metabolic pattern affects not only tumor cells but also other cell types, including T cells [16, 17]. Resting T cells usually express low levels of MCT1 and MCT4 and rely most on OXPHOS to meet minimal energy needs [18, 19]. However, upon antigen stimulation, T cells undergo metabolic reprogramming. They switch from OXPHOS to glycolysis to support activation and produce large amounts of lactate [16, 20–22].

In the TME, activated T cells compete with tumor cells for glucose and also export lactate via MCTs to avoid intracellular acidification. In mouse sarcoma models, highly glycolytic tumor cells outcompete infiltrating T cells for glucose. This metabolic competition weakens T cell mTOR signaling, reduces glycolytic capacity, and impairs effector functions such as IFN-γ production, leading to immune dysfunction. Immune checkpoint blockade can partially reverse these effects by improving glucose availability and restoring T cell metabolism [23]. Under glucose limitation, T cells may use glutamine-dependent anaplerosis to replenish TCA cycle intermediates, providing an alternative way to maintain effector functions [24]. However, high lactate levels restrict this metabolic flexibility, as extracellular lactate from tumor cells blocks normal lactate efflux from activated T cells [25]. This disruption causes lactate to build up inside T cells, impairing their survival, proliferation, and migration [26–30]. Furthermore, excess lactate suppresses pyruvate carboxylase activity and disrupts the TCA cycle, which damages the cytotoxic function of CD8⁺ cytotoxic T lymphocytes (CTLs) and reduces pro-inflammatory cytokine production [31–33].

In recent years, protein lactylation, a new type of post-translational modification (PTM) derived from lactate, has been identified. In this modification, lactyl groups are covalently attached to lysine residues on proteins [34]. There are three known types of lysine lactylation: L-lactyllysine (K_L-la), D-lactyllysine (K_D-la), and N-ε-carboxyethyllysine (Kce), with K_L-la being the most common. This modification plays a key role in regulating gene expression under high glycolysis [35, 36]. Lactylation can modify both histone proteins (e.g., H3K18la) and non-histone proteins, regulating immune responses, tissue repair, and cellular metabolism [37]. Previous studies have shown that lactylation can modulate CD8⁺-T cell metabolism and strongly affect CD4⁺-T cell subset differentiation [38, 39].

Thus, in the TME, lactate acts not only as a metabolic byproduct that causes energetic stress and acidification in T cells but also as an epigenetic regulator through protein lactylation. Together, these effects create a strongly immunosuppressive environment. The sections below explore the specific molecular mechanisms by which lactate impairs T cell function.

## Multilayered regulation of T cell and other immune cell functions by lactate in the TME

### Effects of lactate on CD8^+^-T cells

**Fig. 1:**
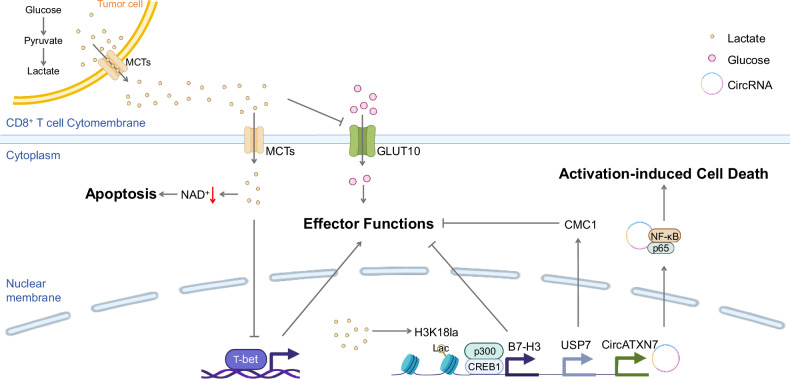
Lactate-mediated molecular mechanisms impairing CD8^+^-T cell anti-tumor function. Excess lactate in the TME impairs CD8⁺-T cell function through multiple pathways: increasing MCT-dependent lactate uptake, causing intracellular lactate buildup and NAD⁺ depletion-driven apoptosis; blocking GLUT10-mediated glucose uptake to inhibit effector function; and reducing T-bet expression. Lactate-driven histone lactylation (e.g., H3K18la) upregulates immune checkpoints such as B7-H3 and increases USP7, which stabilizes CMC1 and suppresses effector function. Lactylated circular ATXN7 sequesters NF-κB p65, promoting AICD. Together, these pathways reduce CD8⁺-T cell cytotoxicity and enable tumor immune evasion.

**Fig. 2:**
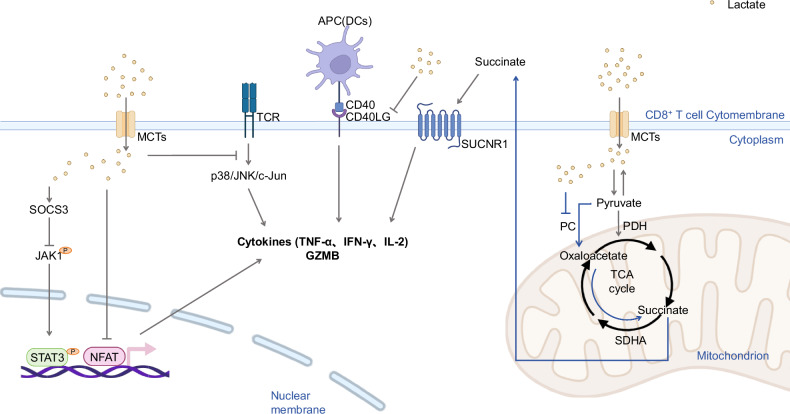
Molecular mechanisms by which lactate inhibits cytokine production and secretion in CD8^+^-T cells. High lactate in the TME suppresses CD8^+^ T-cell production and cytokine secretion by inhibiting the NFAT and p38/JNK/c-Jun pathways and downregulating the CD40LG-JAK/STAT3 pathway. Lactate-induced reduction in extracellular succinate weakens SUCNR1-dependent autocrine activation, lowering cytokines and GZMB secretion.

Lactate impairs the anti-tumor function of CD8⁺-T cells through multiple mechanisms, including disruptions in energy metabolism, effector activity, differentiation, and epigenetic regulation [40]. First, lactate directly affects CD8^+^-T cell effector function by interfering with glucose uptake. GLUT10, a key glucose transporter in CD8⁺-T cells, is directly targeted by lactate accumulated in the TME. Lactate binds to GLUT10 and reduces its glucose transport capacity, which in turn weakens CD8⁺-T cell effector functions [41]. Lactate also inhibits T cell differentiation by reducing the expression of T-bet, a critical transcription factor for CD8⁺-T cell maturation into effector cells [42]. In addition, lactate depletes intracellular nicotinamide adenine dinucleotide (NAD⁺), an essential metabolic coenzyme, leading to T cell apoptosis [43]. Lactate additionally modulates the expression and stability of specific proteins, thereby impairing T cell function. For instance, in the lactate-rich TME, CMC1 expression is enhanced through stabilization and deubiquitination, which contributes to CD8⁺-tumor-infiltrating lymphocytes (TILs) dysfunction by impairing their effector activity (Fig. 1) [44]. Lactate also influences cytokine production in CD8^+^-T cells. It reduces the secretion of key anti-tumor cytokines such as IFN-γ, TNF-α, and IL-2, and disrupts cytotoxic signaling pathways such as NFAT and the p38/JNK/c-Jun axis, directly inhibiting CTL effector function (Fig. 2) [28, 32, 45].

Under normal conditions, cytotoxic T cells use pyruvate carboxylase (PC)-dependent anaplerosis to produce and secrete succinate, which activates SUCNR1 to trigger autocrine pro-inflammatory signaling and strengthen cytotoxicity. In the TME, however, elevated lactate redirects carbon flux toward pyruvate dehydrogenase (PDH) and promotes succinate oxidation instead of secretion. This disrupts SUCNR1-dependent activation, reduces IFN-γ and GZMB secretion, and lowers CD8^+^-T cell effector activity (Fig. 2) [31]. Moreover, exhausted T cells—especially terminal precursor exhausted T cells—upregulate the lactate transporter MCT11 (encoded by SLC16A11), which increases lactate uptake. Higher intracellular lactate worsens T cell dysfunction and exhaustion. Studies in mouse models have shown that knocking out MCT11 or blocking its function with antibodies reduces lactate uptake, restores T cell effector functions, and slows tumor growth. These findings identify MCT11-mediated lactate uptake as a key mechanism of T cell dysfunction in the TME [46]. In sepsis models with high lactate, downregulation of CD40LG and SOCS3 suppresses the JAK-STAT pathway and inhibits T cell activity (Fig. 2) [47]. Furthermore, circDNA2v stabilizes IGF2BP3 and maintains MYC levels to suppress tumor cell senescence and pro-inflammatory factor release. This inhibits CXCL10 and IL-9 mediated T cell infiltration and affects the JAK-STAT3 pathway [48].

In recent years, protein lactylation—especially histone lactylation—has emerged as a key mechanism underlying lactate-driven immunosuppression in the TME. Tumor-specific CD8⁺-T cells from KRAS-mutant tumors show higher global histone lactylation, especially H3K18la, than those from KRAS wild-type tumors. This modification increases expression of circATXN7, a circular RNA that sequesters cytoplasmic NF-κB/p65, making T cells more susceptible to activation-induced cell death (AICD) and impairing function (Fig. 1) [49]. Lactate-induced histone lactylation (H3K18la) also works with the transcription factor CREB1 and its coactivator p300 to bind the B7-H3 promoter and drive its expression. This reduces CD8⁺-T cell infiltration and promotes cancer progression (Fig. 1). Notably, inhibiting key glycolytic enzymes effectively reduces lactate production and histone lactylation, restoring CD8⁺-T cell anti-tumor activity [2].

In summary, lactate compromises CD8⁺-T cell anti-tumor function through metabolic inhibition, disrupted signaling, transcriptional repression, and epigenetic modifications such as lactylation. Together, these processes establish a profoundly immunosuppressive TME.

### Lactate promotes Treg stability and immunosuppressive function

Lactate accumulation in the TME has emerged as a critical regulator of immune responses, especially by modulating Treg function. Studies show that lactate in the TME preferentially drives naive CD4⁺-T cells to become Tregs while suppressing Th1 and Th17 development, creating an immunosuppressive environment that supports tumor progression [50]. Several mechanisms contribute to this effect. First, lactate impairs Th1 polarization by promoting SIRT1-mediated deacetylation and degradation of T-bet, the master transcription factor for Th1 differentiation [50]. Second, lactate boosts LDHA activity in naive CD4⁺-T cells, promoting the conversion of α-ketoglutarate (α-KG) to 2-hydroxyglutarate (2HG). Accumulated 2HG inhibits ATP5B, activates mTOR signaling, suppresses HIF-1α synthesis, and stabilizes Foxp3, favoring Treg differentiation while blocking Th17 development [51]. In addition, lactate induces epigenetic reprogramming in Th17 cells through histone H3K18 lactylation. This reduces IL-17 production and enhances Foxp3 expression through IL-2 signaling, effectively converting Th17 cells into Tregs [52]. Lactate also triggers metabolic reprogramming in Tregs by promoting lactylation of MOESIN at Lys72. This modification improves Treg retention in the TME by strengthening interaction TGF-β receptor I and enhancing downstream SMAD3 signaling [53].

**Fig. 3:**
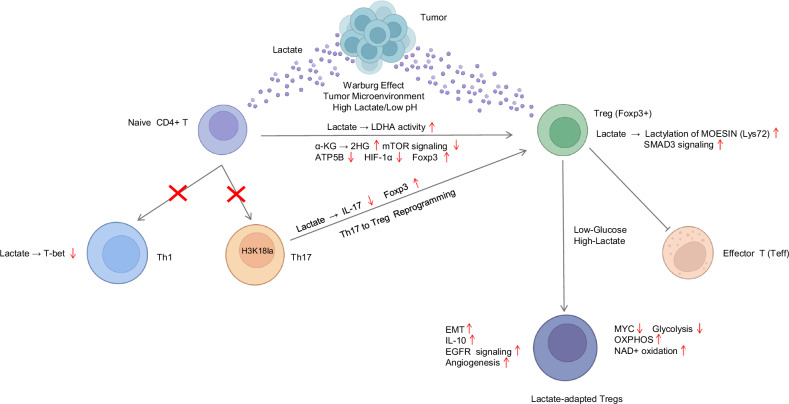
Mechanisms of lactate-driven Treg differentiation, metabolic reprogramming, and enhanced immunosuppression in the TME. Regulation of T Cell Subset Differentiation: Th1 suppression: Lactate promotes T-bet degradation, inhibiting Th1 polarization. Treg Promotion: Lactate activates LDHA in naive CD4⁺-T cells, favoring Treg differentiation while suppressing Th17 development. Th17 to Treg conversion: Lactate induces H3K18 lactylation in Th17 cells, reprogramming them into Tregs. Treg metabolic enhancement: Lactate increases Foxp3 expression, boosting OXPHOS and improving Treg metabolic fitness and survival. Treg retention: MOESIN lactylation(Lys72) enhances SMAD3 signaling, promoting Treg retention and immunosuppressive function. Collectively, these mechanisms strengthen Treg immunosuppression, weaken anti-tumor immunity, and drive tumor immune evasion, angiogenesis, and growth.

High lactate levels in the TME are strongly linked to elevated Treg suppressive function. In lactate-rich environments, Tregs show superior immunosuppressive activity due to their unique metabolic flexibility. Unlike most effector T cells, which rely heavily on glycolysis, Tregs preferentially use lactate as an energy source for proliferation. They efficiently take up lactate to support the TCA cycle and gluconeogenesis, maintaining proliferation and suppressive function[54]. Lactate not only drives Treg differentiation but also enhances their function. High lactate in the TME increases Foxp3 expression, the defining transcription factor of Tregs. Foxp3 suppresses MYC and glycolysis, enhances OXPHOS, and promotes NAD⁺-oxidation, reprogramming Treg metabolism. These Foxp3-mediated changes give Tregs a survival advantage in low-glucose, lactate-rich environments, enabling them to resist lactate-induced suppression of effector T cell proliferation and function. As a result, Tregs help build an immunosuppressive niche that enables tumor immune evasion [55]. Watson et al. identified two metabolically flexible Treg subsets: one dependent on glucose, the other on lactate. Lactate-using Tregs exhibited stronger immunosuppressive and pro-tumor activity than glucose-using Tregs. These lactate-adapted Tregs secreted immunosuppressive cytokines such as IL-10 and promoted EMT, EGFR signaling, and angiogenesis. Under nutrient-deprived conditions, they maintained high metabolic activity by using lactate, further enhancing their tumor-promoting ability[56]. The aforementioned mechanisms are illustrated in Fig. 3.

Thus, targeting lactate metabolism is a promising therapeutic strategy to disrupt Treg-mediated immunosuppression and improve cancer immunotherapy. By modulating Treg activity in the lactate-rich TME, new approaches can be developed to enhance immune checkpoint blockade and other immunotherapies.

### Lactate indirectly regulates T cell function via other immune cells

#### Regulation of dendritic cell (DC) function by lactate

**Fig. 4:**
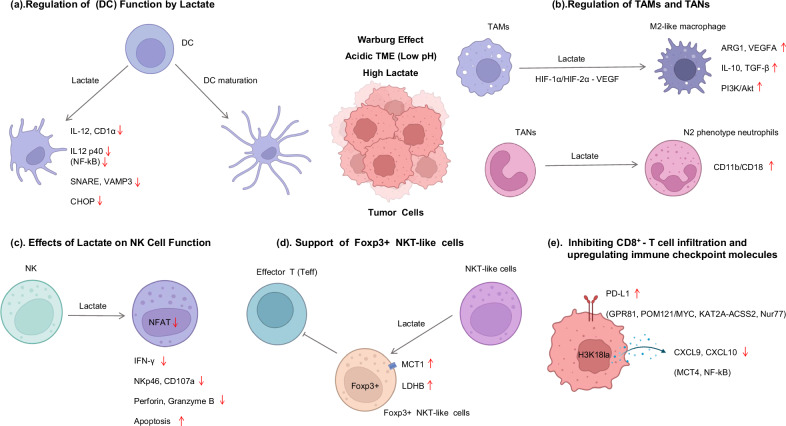
Lactate in the TME suppresses CD8^+^-T cells by acting on multiple cell types. **a** Lactate impairs DC function, inhibiting activation and antigen presentation. **b** Lactate modulates TAMs and TANs, promoting immunosuppressive phenotypes. **c** Lactate suppresses NK cell cytotoxicity and induces apoptosis. **d** Lactate acts on NKT-like cells, reducing anti-tumor activity. **e** Lactate inhibits CD8⁺-T cell infiltration and upregulates immune checkpoints to immune evasion. Collectively, these lactate-driven effects across multiple cell types synergistically suppress CD8⁺-T cell function and enable tumor immune escape.

Lactate, a key metabolic byproduct in the TME, strongly affects DC function, particularly by impairing antigen presentation and T cell priming. Studies show that lactate in tumors suppresses multiple DC processes needed to activate effective effector T cell responses, including differentiation, maturation, activation, and antigen processing, transport, and presentation. Both lactate and low pH in the TME promote monocyte-to-DC differentiation by enhancing mitochondrial respiration and inhibiting the mTOR pathway, a major nutrient-sensing signaling axis [57]. Lactate reduces the DNA-binding ability of NF-κB, p65, and c-Rel to the IL-12p40 promoter, blocking DC expression of IL-12p40 and downstream Th1 responses [58–61]. Lactate also hinders the development of IL-12-producing CD1a⁺ DCs [62]. High lactate levels inhibit DC differentiation and maturation. In the presence of tumor-derived interleukin-4 (IL-4) and granulocyte-macrophage colony-stimulating factor (GM-CSF), lactate suppressed CD1a expression independent of pH. Consequently, DC precursors fail to express CD1a, a marker required for functional DC maturation [58]. Acidic pH also disrupts peptide-MHC class I interactions, which is particularly harmful for cross-presentation, a key step for priming CD8⁺-T cells [63]. In melanoma models, the lactate–SREBP2 (sterol regulatory element-binding protein 2) axis disrupts cross-presentation. Mechanistically, lactate downregulates key proteins involved in endosome-lysosome fusion and post-Golgi trafficking, including vesicle-associated membrane protein 3 (VAMP3), syntaxin 6 (Stx6), and Rab27, impairing cross-presentation [64]. Lactate also regulates ER stress through a C/EBP homologous protein (CHOP)-dependent pathway, blocking lysosome formation and disrupting effective antigen presentation by DCs [65]. Lactate suppresses MHC class II expression both indirectly (via EGR1 and SRF) [59] and directly (through GPR81 signaling) [66]. Together, these mechanisms impair DC-mediated presentation of tumor-specific antigens. GPR81-dependent lactate signaling also reduces IFN-α production by plasmacytoid DCs (pDCs) in tumors [67]. In patients with metastatic melanoma, lactic acidosis has been suggested to suppress secretion of cytokines, such as IFN-α and CXCL10 by circulating pDCs upon Toll-like receptor 7 (TLR7) and 8 (TLR8) stimulation [68]. The aforementioned mechanisms are illustrated in Fig. 4a.

In summary, lactate exerts complex, multifaceted regulatory effects on DCs. By inhibiting DC maturation and antigen presentation, lactate reduces T cell activation and promotes immune tolerance in the TME. These mechanisms highlight a new therapeutic approach: targeting lactate metabolism or its downstream signaling may improve immunotherapy and overcome tumor immune evasion.

#### Regulation of tumor-associated macrophages (TAMs) and tumor-associated neutrophils (TANs) by lactate

In the TME, lactate accumulation is strongly associated with the polarization of TAMs toward an M2-like, pro-tumor phenotype, which exacerbates the immunosuppressive environment and supports tumor progression. In 2014, Colegio et al. demonstrated that tumor-derived lactate promotes M2-like macrophage polarization through the HIF-1α–VEGF axis [69]. Lactate also drives pro-tumor effects via the ATP6V0D2-HIF-2α-VEGF pathway [70]. High lactate levels drive TAMs towards an M2-like phenotype characterized by arginase 1 (ARG1), VEGFA, and anti-inflammatory cytokines such as IL-10 and TGF-β, all of which support tumor growth and immune suppression [69, 71–75]. Lactate can also stimulate pro-inflammatory macrophages in vitro. Studies in male mice with gut-specific Slc16a1 knockout show reduced intestinal macrophages and lower interstitial lactate levels, suggesting a close link between lactate transport and macrophage homeostasis [76]. Studies in KRAS-mutant lung adenocarcinoma and LKB1-deficient tumors further support lactate’s role in macrophage polarization. These tumors produce and export more lactate, which promotes M2-like polarization and T cell dysfunction *via* MCT4 and the lactate receptor GPR81 (also known as hydroxycarboxylic acid receptor 1, HCAR1) on immune cells [77]. Interestingly, TAMs exhibit high glycolytic activity and lactate secretion similar to M1-like macrophages, but this metabolism is required for maintaining the expression of M2/M(IL-4) markers, inhibiting glycolysis significantly downregulates these markers [75]. Lactate also activates receptors such as Axl and Tie2, driving macrophages towards an immunosuppressive, pro-angiogenic phenotype that supports tumor growth [78]. In many cancers, higher M2-like macrophage abundance correlates with worse patient prognosis, underscoring lactate’s central role in tumor progression and immune evasion.

Tumor-associated neutrophils (TANs) are another key immunosuppressive component in the TME, and lactate is a major driver of their activation. Lactate promotes neutrophil polarization toward a tumor-supportive N2 phenotype that enhances tumor invasion and metastasis [79, 80]. In acidic TMEs, TANs show higher expression of β2 integrins and CD11b/CD18, contributing to their pro-tumor activity. TANs promote cancer progression and spread by suppressing T-cell responses, producing angiogenic factors, and secreting proteolytic enzymes such as MMP-9 and elastase. These factors drive ECM remodeling, immune evasion, and tumor invasion [79]. Lactate also expands myeloid-derived suppressor cells (MDSCs) and enhances their ability to suppress T cell function, further worsening tumor immune escape [81–83]. The mechanisms above are illustrated in Fig. 4b.

#### Effects of lactate on NK cell function

Natural killer (NK) cells are critical for antitumor immunity. They directly kill cancer cells by releasing cytotoxic granules and cytokines, and they coordinate broader immune responses. However, the immunosuppressive TME severely impairs NK cell function, lactate accumulation is a major cause. Husain et al. demonstrated that lowering local pH from 6.8 to 6.0 drastically reduces NK cell activity and cytotoxicity against target cells [84]. Mechanistically, lactate acts through multiple pathways. Brand et al. [28] reported that pathophysiological lactate levels block the upregulation of the nuclear factor of activated T cells (NFAT) in both T cells and NK cells, reducing interferon-gamma (IFN-γ) production and suppressing lymphocyte activation. Lactate concentrations above 20 mM can also directly induce NK cell apoptosis, which may explain lower lymphocyte infiltration in high-lactate tumors. Harmon et al. [85] showed that in colorectal liver metastases, tumor-derived lactate acidifies the TME. Liver-resident NK cells migrate to these sites cannot regulate intracellular acidification, leading to mitochondrial stress and apoptosis. Husain et al. [82] also found that high lactate downregulates activating receptors such as NKp46 and the degranulation marker CD107a on NK cells. This reduces perforin and granzyme B expression, rendering NK cells inactive and enabling immune evasion. Targeting lactate transport represents a promising strategy to restore NK cell function. In breast cancer models, Long et al [86]. showed that blocking MCT4-mediated lactate efflux reverses TME. This increases expression of the activating receptor NKG2D and its ligand H60a, ultimately improving NK cell-mediated tumor control. The aforementioned mechanisms are illustrated in Fig. 4c.

#### Effects of lactate on natural killer T (NKT)-like cells

Lactate is a critical metabolic signal in the TME, and growing evidence highlights its role in regulating immune cell function. Wang et al. identified a unique FOXP3⁺ NKT-like cell population in the TME of malignant pleural effusions (MPE) [87], a functionally “cold” immune environment. Flow cytometry and immunofluorescence confirmed a distinct FOXP3^+^ NKT-like subset in MPE. Single-cell RNA sequencing showed that glycolytic and pyruvate metabolism pathways are significantly upregulated in these cells. Chemical or genetic disruption of lactate metabolism reprograms the epigenome and transcriptome of NKT-like cells toward a Treg-like state in the high-lactate MPE microenvironment. Similar to Tregs, these cells highly express MCT1 and LDHB(an enzyme involved in lactate metabolism) which supports lactate uptake and utilization. These processes are essential for maintaining their immunosuppressive phenotype and the high levels of protein lactylation observed in MPE. The mechanisms as mentioned earlier are illustrated in Fig. 4d.

### Lactate promotes tumor immune evasion by inhibiting CD8^+^-T cell infiltration and upregulating immune checkpoint molecules

In hepatocellular carcinoma (HCC), elevated MCT4 expression increases lactate efflux and TME acidification. This impairs CD8⁺-T cell infiltration and cytotoxicity, weakening anti-tumor immunity. Pharmacological or genetic inhibition of MCT4(e.g., with VB124) reduces TME acidification, increases intracellular ROS, and activates NF-κB signaling. Activated NF-κB induces secretion of CXCL9 and CXCL10, boosting CD8⁺-T cell recruitment and effector function [88]. In glioblastoma, high intratumoral lactate levels hinder CD8⁺-T cell migration and infiltration, contributing to immune suppression [89].

Lactate also promotes tumor immune evasion by upregulating PD-L1 expression on tumor cells through multiple mechanisms. For example, PRMT3 enhances lactate-induced PD-L1 expression by increasing H3K18la binding to PD-L1 promoter. Anti-PD-L1 therapy reverses PRMT3-driven tumor growth and restores CD8^+^-T cell infiltration [90]. Lactate also acts as a signaling molecule in tumor progression, angiogenesis promotion, immune evasion, and chemoresistance across cancers. GPR81 (HCAR1), a lactate-sensing GPCR, binds lactate and triggers intracellular signaling. For example, lactate signaling through GPR81 increases PD-L1 expression, suppressing CD8⁺-T cell cytotoxicity and promoting immune tolerance in the TME [91]. Lactate also supports the TME by enhancing ECM remodeling and invasiveness [92]. In addition, H3K18la directly activates the transcription of POM121, which facilitates MYC nuclear translocation and binding to the CD274 promoter, inducing PD-L1 expression. This leads to PD-L1 overexpression and immune evasion in non-small cell lung cancer (NSCLC) [93]. In glioblastoma, the acetyltransferase KAT2A interacts with ACSS2(a lactyl-CoA-producing enzyme) to form a complex that mediates H3 lactylation, upregulates PD-L1, and promotes tumor immune evasion [94]. In HCC, high MCT expression in Treg cells and lactate accumulation are linked to poor prognosis and resistance to PD-1 therapy [95]. In preclinical small cell lung cancer (SCLC) models, lactate inhibition reduces Nur77 expression, restores T cell function, and enhances PD-1 blockade efficacy, reducing tumor burden and extending survival [96]. The aforementioned mechanisms are illustrated in Fig. 4e.

## Tumor immunotherapeutic strategies targeting lactate metabolism

Emerging research on lactylation and related enzymes suggests that targeting protein lactylation holds great promise for boosting immune cell anti-tumor function and inhibiting tumor progression [97]. These insights reveal novel therapeutic targets for anti-cancer drug development. Therapies focused on lactylation may target key proteins in lactate production, transport, or lactylation-modifying enzymes. Current investigation therefore focuses on inhibitors of enzymes and transporters in lactate metabolism.

### Development and application of LDHA inhibitors

Inhibiting key enzymes in lactate metabolism reduces lactate production and thereby suppresses lactylation. LDHA is a critical glycolytic enzyme that converts pyruvate to lactate. It is often overexpressed in many cancers and associated with poor prognosis. Several potent LDHA inhibitors have been developed. Oxamate, a pyruvate analog, acts as a competitive LDHA inhibitor and suppresses tumor cell proliferation [98]. NADH-competitive inhibitors such as Gossypol (AT-101), FX11 (3-dihydroxy-6-methyl-7-(phenylmethyl)-4-propylnaphthalene-1-carboxylic acid), and quinoline-3-sulfonamides also block tumor cell growth [99–101]. Galloflavin directly binds and inhibits LDHA, reducing lactate production and inducing tumor cell apoptosis [102]. However, broad off-target effects and complex interactions with cellular pathways challenge clinical translation. Non-specific LDHA inhibition may cause unpredictable and harmful side effects, limiting clinical application.

Dichloroacetate (DCA), an orally bioavailable small molecule, inhibits pyruvate dehydrogenase kinase (PDK), shifting metabolism from glycolysis to OXPHOS. This switch promotes mitochondria-mediated apoptosis and suppresses tumor proliferation. DCA has shown therapeutic potential in glioblastoma and advanced head and neck squamous cell carcinoma [103–105].

Inhibitors targeting lactate metabolism affect not only intracellular lactylation but also entire cellular metabolic pathways. Future drug development should aim to improve specificity, minimize side effects, and precisely characterize effects on site-specific lactylation. Overcoming these challenges is essential to translate these inhibitors into clinically useful therapeutics targeting lactylation (Table 1).

**Table 1:** Drugs targeting lactate production and transport.

Molecule	Taget	Mechanism	Condition	Clinical trial
Oxamate	LDH	Inhibits LDH-A	N/A	N/A
AT-101 (gossypol)	LDH	Inhibits LDH	Malignant tumor	Phase ll Trial
FX-11	LDH	Inhibits LDH-A	Pancreatic cancer	N/A
Quinoline 3-sulfonamides (GSK2837808A)	LDH	Inhibits LDH-A	N/A	N/A
Galloflavin	LDH	Binds the free enzyme	Malignant tumor	N/A
DCA	PDK	Increases glucose uptake into mitochondria	Malignant tumor,Lactic acidosis	Phase II/III Trial
Lonidamine	HK	Inhibits glycolysis	Malignant tumor	Phase II Trial
CHC(α-Cyano-4-hydroxycinnamate)	Lactate transporters	Inhibits MCT1/2	Hyperglycolytic malignancies	N/A
Quercetin	Lactate transporters	Inhibits MCT1/2	N/A	N/A
DIDS (4,4′-diisothiocyanatostilbene-2,2′-disulphonic acid)	Lactate transporters	Inhibits MCT1	N/A	N/A
Phloretin	Lactate transporters	Inhibits MCT1/2/4	N/A	N/A
P-chloromercuribenzenesulphonate	Lactate transporters	Inhibits MCT	N/A	N/A
AR-C155858	Lactate transporters	Inhibits MCT1/2	Hyperglycolytic malignancies	N/A
AZD3965	Lactate transporters	Inhibits MCT1/2	Malignant tumor	Phase I Trial
BAY-8002	Lactate transporters	Inhibits MCT1	N/A	N/A
Syrosingopine	Lactate transporters	Inhibits MCT1/4	Malignant tumor	N/A

*N/A* not available.

### Monocarboxylate transporter (MCT) inhibitors

Lactate is produced intracellularly and transported between cells *via* MCTs, which regulate many physiological and pathological processes. Tumor lactate metabolism strongly depends on MCT4-mediated efflux and MCT1-mediated influx. Targeting MCT1 and MCT4 is therefore a promising strategy, especially for chemotherapy-resistant cancers[106]. Several MCT inhibitors have been identified, including α-cyano-4-hydroxycinnamate (CHC), phloretin, p-chloromercuribenzenesulphonate, 4,4’-diisothiocyanatostilbene-2,2’-disulphonic acid (DIDS), lonidamine, and quercetin. These compounds show therapeutic potential across tumor types [107, 108]. Lonidamine, an indazole-3-carboxylic acid derivative, shows promise in suppressing liver cancer stem cell oncogenicity by inhibiting histone H3K9 and H3K56 lactylation. It is also being tested in combination with standard chemotherapy for solid tumors [109, 110]. Additionally, more selective small-molecule MCT inhibitors include AR-C155858, AZD3965, and BAY-8002, which potently inhibit MCT1 and modulate immunity. A Phase I clinical trial reported preliminary safety and efficacy of AZD3965 in patients with advanced solid tumors and lymphomas [111–113]. Moving forward, a deeper understanding of the specific roles of different MCTs in cancer, their regulation of lactylation, and their structural biology will be critical. This knowledge will support the development of highly selective MCT inhibitors for clinical use (Table 1).

Lactate-mediated drug resistance is a major challenge in cancer therapy. Combination strategies offer a promising solution, including for cancer immunotherapy. Given the close link between lactate metabolism and tumor immunity, combining lactate-targeted therapies with immunotherapy has become a novel therapeutic paradigm. For example, lactate upregulates PD-1 on Tregs [114]. Combining lactate-reduction with PD-1 inhibitors may therefore improve efficacy. Similarly, pairing LDHA or MCT inhibitors (which reduce lactate production or protumor effect) with immune checkpoint inhibitors (ICIs) could greatly improve immunotherapy outcomes. Simultaneously targeting multiple pathways to boost tumor immunotherapy is thus a feasible and promising direction.

### Targeting lactylation in tumor immunotherapy

Beyond metabolic regulation, lactylation—a novel PTM that adds lactyl groups to protein lysines—plays a critical role in anti-tumor immunity and immunotherapy response. PD-1 blockade efficacy depends on the balance between PD-1-expressing CD8⁺-T cells and Tregs in the TME. Gu et al. showed that lactate promotes tumor progression by modulating MOESIN lactylation in Treg, enhancing their immunosuppressive function. Notably, Tregs from HCC patients who responded well to anti-PD-1 therapy had significantly lower lactylation levels, suggesting lactylation could serve as a predictive biomarker or therapeutic target [53].

Kumagai et al. reported that in highly glycolytic tumors, Tregs rapidly take up lactate via MCT1, promoting NFAT1 nuclear translocation and PD-1 upregulation. In contrast, high lactate suppresses PD-1 on effector T cells, leading to immune escape and therapy resistance [114]. Lactate also induces PD-L1 on macrophages and neutrophils, further contributing to immune resistance[115]. Weng et al. identified that targeting the MCT1/miR-34a/IL-6/IL-6R axis suppresses M2 macrophage polarization in triple-negative breast cancer, potentially reversing immunosuppression[116]. Preclinical studies show that combining the MCT1 inhibitor AZD3965 with anti-PD-1 therapy reduces TME lactate and enhances anti-tumor immunity [117]. Similarly, LDHA inhibitors plus PD-1 blockade produce stronger anti-tumor effects than PD-1 inhibition alone. These findings suggest that inhibiting lactylation-related processes alongside immunotherapy may represent a novel, synergistic cancer treatment (Table 1).

Progress in understanding lactate metabolism and protein lactylation has established these processes as promising therapeutic targets. Exploring the regulatory sites and functional outcomes of lactylation will help identify effective therapeutic targets and guide new combination strategies. However, despite strong preclinical and clinical evidence supporting lactate as a target to boost anti-tumor immunity and sensitize tumors to therapy, the precise mechanisms and biological roles of lactylation remain incompletely understood. Most current interventions inhibit lactate production, transport, or downstream signaling, but their specificity and efficacy for modulating lactylation need major improvement. Future research should identify and characterize lactylation-specific “writers”, “erasers”, and “readers”, similar to enzymes in other epigenetic modifications. These proteins will enable direct, selective targeting of lactylation and may open new avenues for tumor immunotherapy.

## The function of lactate in non-tumor immunity

Although lactate is widely regarded as an immunosuppressive metabolite in the TME, accumulating evidence shows its immune effects are highly context-dependent. In autoimmune and chronic inflammatory diseases, lactate can instead promote effector immune responses. Serum lactate levels are elevated in rheumatoid arthritis (RA) patients, promoting CD4^+^T cell activation, IL-21 secretion, and higher proportions of Th17 and Tph cells [118]. In Sjögren’s syndrome, lactate accumulates in inflamed tissues and exerts key immunoregulatory effects. On one hand, lactate enters CD4⁺ T cells via SLC5A12, increasing IL-21 expression and driving tertiary lymphoid structures (TLS) formation, enhancing local adaptive immunity. On the other hand, abnormal lactate buildup in acinar epithelial cells causes mitochondrial dysfunction, mtDNA damage, and cytoplasmic leakage, activating the cGAS-STING pathway. Sustained activation amplifies NF-κB and type I interferon signaling and strongly boosts pro-inflammatory cytokines including IL-6, IL-8, IFN-α, IFN-β, and TNF-α [119, 120]. In chronic kidney disease (CKD), lactate enhances inflammation and fibrosis by promoting histone H4K12 lactylation (H4K12la) and activating NF-κB signaling, suggesting lactate metabolism is a key mechanism in CKD progression [121]. Beyond autoimmunity, lactate-mediated histone lactylation contributes to trained innate immunity after BCG vaccination. In humans vaccinated with BCG, increased lactate production and sustained histone lactylation (e.g., H3K18la) in monocytes/macrophages are associated with enhanced transcriptional responses and higher pro-inflammatory cytokine production (TNF, IL-6) upon stimulation, linking metabolic rewiring with epigenetic memory in trained immunity [122].

Beyond immune modulation in autoimmunity and infection, excess lactate may also contribute to systemic metabolic disorders. Recent studies demonstrate that circulating lactate levels positively correlate with cancer-associated cachexia and metabolic wasting. Mechanistically, tumor-derived lactate acts on peripheral tissues to promote adipose remodeling, muscle catabolism, and energy imbalance, driving systemic weight loss and metabolic dysfunction [123]. Dysregulated glucose metabolism and enhanced Cori cycle activity—where the liver recycles tumor lactate into glucose—further increase whole-body energy expenditure and insulin resistance in cancer cachexia [124]. These findings indicate lactate acts not only locally in the TME but also as a circulating signal that coordinates tumor growth with systemic metabolic remodeling.

These findings highlight the pleiotropic nature of lactate as a metabolic immune regulator. Systemic targeting of lactate metabolism in cancer may therefore have unintended consequences, especially in patients with pre-existing autoimmune disease or trained immunity. Such context-dependent effects may contribute to immune-related adverse events during checkpoint blockade, warranting careful patient stratification and mechanistic evaluation in future therapies.

## Conclusion

**Fig. 5:**
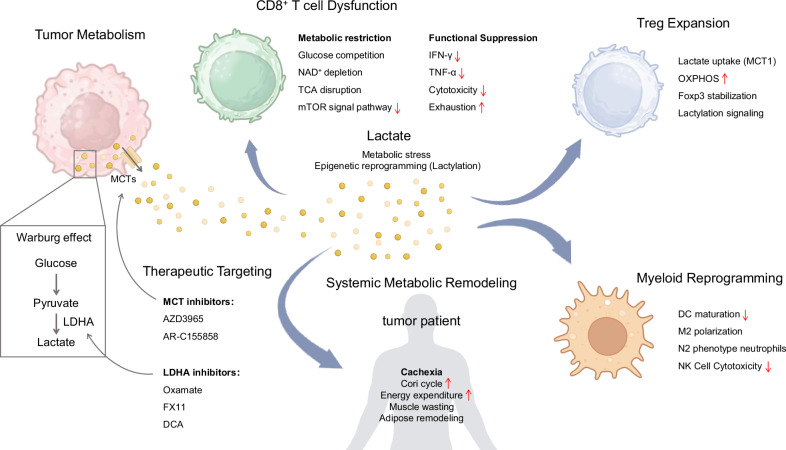
Lactate-mediated immunosuppression and systemic metabolic dysfunction in cancer. Tumor cells produce lactate via the Warburg effect and secrete it into the microenvironment through MCT transporters. Extracellular lactate impairs CD8⁺-T cell cytotoxic function, expands immunosuppressive Tregs, and reprograms myeloid cells toward a pro-tumor phenotype, while driving systemic cachexia. Targeting LDHA (lactate synthesis) or MCTs (lactate transport) is a promising strategy to reverse tumor immunosuppression and metabolic dysfunction.

In the TME, lactate has emerged as a pivotal metabolic player with an increasingly recognized role in tumor immune evasion. Lactate accumulation suppresses T cell-mediated anti-tumor immunity through multiple mechanisms: inhibiting T cell proliferation and cytokine production, while promoting immunosuppressive cell populations. These effects enable tumors to escape host immune surveillance. Research indicates that lactate not only directly impairs CD8⁺-T cell metabolism and effector function but also activates Tregs and immunosuppressive myeloid cells, reshaping the TME immune landscape and driving systemic cachexia in cancer patients. Therapeutic targeting of LDHA or MCTs represents a promising strategy to reverse tumor immunosuppression and metabolic dysfunction. This complexity reveals lactate as a multifaceted immune regulator. The aforementioned mechanisms are illustrated in Fig. 5.

In recent years, enzymes and transporters in lactate metabolism have emerged as novel targets for modulating tumor immunity. Inhibitors targeting these molecules have shown promising therapeutic efficacy in preclinical tumor models, supporting their clinical potential. These studies not only open new therapeutic avenues, but also deepen our understanding of the TME. However, lactate’s immune effects vary across tumor types, so future research must clarify these context-specific mechanisms to advance precision tumor immunotherapy. Combining immune checkpoint inhibitors with metabolic interventions holds great promise for overcoming lactate-driven immunosuppression. This multi-target approach addresses monotherapy limitations and improves overall immunotherapy efficacy. By integrating metabolic modulation and immune activation, T cell anti-tumor activity can be effectively enhanced, strengthening systemic immunity. Emerging studies also highlight the regulatory roles of lactate and histone lactylation on non-cellular TME components such as the ECM which can significantly influence immune evasion and therapy resistance [103, 105, 125, 126].

In some cancers, lactate can also suppress CD8⁺-T cells through non-immune cells. In diabetes-associated pancreatic cancer, tumor-associated Schwann cells (TASs) with a “METTL16^+^ CD276^+^ NECTIN2^+^^”^ phenotype are increased and closely linked to CD8⁺-T cell exhaustion. Mechanistically, high lactate is taken up by Schwann cells via MCT1/MCT4. Intracellular lactate interacts with the RNA methyltransferase METTL16, altering its modification state, stabilizing specific transcription factors, and driving upregulation of the immunosuppressive molecules CD276 and NECTIN2. This cascade directly impairs CD8⁺-T cell cytotoxicity and reduces anti-PD-1 efficacy [127].

Notably, some studies report contrasting effects, while most emphasize lactate’s immunosuppressive role, others suggest lactate can enhance immune responses under certain conditions. For example, Feng et al. demonstrated lactate increases CD8⁺-T cell stemness and boosts anti-tumor immunity. Specifically, lactate inhibits HDACs in CD8⁺-T cells, promoting their differentiation into potent cytotoxic cells [128]. Raychaudhuri et al. identified histone H3K18la and H3K9la (lactylation) as key transcriptional initiators in CD8⁺-T cells that enhance expression of genes critical for activation and anti-tumor function. However, the precise mechanisms by which histone lactylation regulates CD8⁺-T cell activation remain incompletely understood [129]. Integrating these diverse findings yields a more complete view of lactate’s dual role in tumor immunity—both immunosuppressive and immunostimulatory—providing a strong theoretical foundation for clinical translation.

Although targeting lactate metabolism is a promising cancer therapy supported by robust preclinical evidence, clinical translation faces major challenges.

- Lactate’s biological role is far more complex than previously appreciated. It suppresses anti-tumor immunity but also supports normal physiology in the heart, skeletal muscle, and brain. Too low a dose may not work, too high may cause off-target toxicity.
- There are still no robust biomarkers to identify patients who are most likely to benefit. In vivo detection and quantification of lactylation remain immature.
- Tumors rapidly reprogram metabolism when glycolysis is blocked, activating glutaminolysis or fatty acid oxidation to compensate, leading to therapeutic resistance.
- Lactate’s function varies with tumor stage and context, requiring stage‑specific therapeutic strategies.
- Large‑scale, prospective clinical trials validating efficacy and safety of lactate‑targeting agents are still lacking.

In conclusion, lactate, a major metabolic product of tumors, plays a complex and multifaceted role in immune evasion. Future research should continue to explore lactate’s context-dependent effects across cancers and focus on combining metabolic pathway targeting with immune checkpoint blockade to accelerate clinical translation of precision cancer immunotherapies. This approach will not only enable more effective cancer treatments but also deepen our understanding of tumor immune escape mechanisms.
